# A German study on the impact of stress on interparental relationship strain after preterm birth

**DOI:** 10.1093/eurpub/ckac130.204

**Published:** 2022-10-25

**Authors:** A Reimer, L Mause, J Hoffmann, A Hagemeier, N Scholten

**Affiliations:** 1 IMVR, University Hospital Cologne, Cologne, Germany; 2 IMSB, University of Cologne, Cologne, Germany

## Abstract

**Background:**

Relationship satisfaction is an important predictor of well-being. Few studies address the effects of stress on interparental relationships of parents with preterm infants. However, the experience of a preterm birth represents an extreme, stressful event and therefore may place a strain on a relationship. Our aim is to examine the impact of postnatal stress on maternal and paternal perceptions of relationship strain.

**Methods:**

As part of the Neo-CamCare project, a retrospective cross-sectional study was conducted targeting parents with infants with a birth weight below 1,500 g. Linear regression was used to analyse the influence of stress on relationship strain.

**Results:**

437 mothers and 301 fathers participated. Data indicate that interparental relationship strain experienced by fathers (M = 2.61, SD = 1.46) is lower than strain experienced by mothers (M = 3.43, SD = 1.7). The stress level due to the infant's behaviour and appearance is lower in fathers (M = 2.53, SD = 0.95) than in mothers (M = 2.98, SD = 1.05). Stress due to parental role change is higher in mothers (M = 3.37, p = 1.04) than in fathers (M = 2.49, SD = 0.99). Regression analyses show that stress due to behaviour and appearance, as well as parental role change, can be associated with relationship strain in mothers. For fathers, only stress experienced due to the behaviour and appearance can be associated with relationship strain, whereas parental role change is not significant.

**Conclusions:**

Our data illustrate that relationship strain can result from stress in mothers and fathers, indicating the need for stress prevention measures for both. Only mothers show relationship strain due to stress in their parental role. Although it is unclear what mechanisms underlie these findings, we assume that the maternal role is still primarily associated with child care. One way to relieve maternal stress could be to increase psychological support and the promotion paternal involvement in the postnatal period.

**Key messages:**

- Understanding the extent to which stress affects relationships helps to address mothers and father equally.
- Based on the findings, parent-centred interventions can be developed to manage relationship strains.

